# Exploring the pathology of an epidermal disease affecting a circum-Antarctic sea star

**DOI:** 10.1038/s41598-018-29684-0

**Published:** 2018-07-27

**Authors:** Laura Núñez-Pons, Thierry M. Work, Carlos Angulo-Preckler, Juan Moles, Conxita Avila

**Affiliations:** 1 Section Biology and Evolution of Marine Organisms (BEOM), Stazione Zoologica Anton Dohrn (SZN), Villa Comunale, 80121 Napoli, Italy; 2 Smithsonian Tropical Research Institute (STRI), Tupper/Naos/Bocas del Toro Labs, Ancón, 0843-03092 Panamá City, Republic of Panama; 3 US Geological Survey, National Wildlife Health Center, Honolulu Field Station, Honolulu, HI 96850 USA; 4 Department of Evolutionary Biology Ecology and Environmental Sciences, and Biodiversity Research Institute (IrBIO), University of Barcelona, Faculty of Biology, Av. Diagonal 643, 08028 Barcelona, Catalonia Spain; 5 Museum of Comparative Zoology & Department of Organismic and Evolutionary Biology, Harvard University, 26 Oxford Street, Cambridge, MA 02138 USA

## Abstract

Over the past decade, unusual mortality outbreaks have decimated echinoderm populations over broad geographic regions, raising awareness globally of the importance of investigating such events. Echinoderms are key components of marine benthos for top-down and bottom-up regulations of plants and animals; population declines of these individuals can have significant ecosystem-wide effects. Here we describe the first case study of an outbreak affecting Antarctic echinoderms and consisting of an ulcerative epidermal disease affecting ~10% of the population of the keystone asteroid predator *Odontaster validus* at Deception Island, Antarctica. This event was first detected in the Austral summer 2012–2013, coinciding with unprecedented high seawater temperatures and increased seismicity. Histological analyses revealed epidermal ulceration, inflammation, and necrosis in diseased animals. Bacterial and fungal alpha diversity was consistently lower and of different composition in lesioned versus unaffected tissues (32.87% and 16.94% shared bacterial and fungal operational taxonomic units OTUs respectively). The microbiome of healthy stars was more consistent across individuals than in diseased specimens suggesting microbial dysbiosis, especially in the lesion fronts. Because these microbes were not associated with tissue damage at the microscopic level, their contribution to the development of epidermal lesions remains unclear. Our study reveals that disease events are reaching echinoderms as far as the polar regions thereby highlighting the need to develop a greater understanding of the microbiology and physiology of marine diseases and ecosystems health, especially in the era of global warming.

## Introduction

Emerging diseases in marine invertebrates seem to be increasing in prevalence, complexity, and sometimes virulence, concomitant with global climate change^1–5^. In addition to their direct impact on affected species, epizootics in marine invertebrates can have important ecological ramifications. For example, a mass die off of sea urchins in the Caribbean led to profound ecosystem shifts manifested by overgrowth of algae and subsequent secular declines in hermatypic corals^6^. The impact of marine diseases, especially in isolated regions, is difficult to investigate because of limited opportunities for long-term observations, especially if the immediate effects are sublethal or can be confounded by other ecological interactions (e.g., competition, predation)^5^. In other cases, epizootics occur too rapidly to be investigated or are not investigated at all^7,8^. Conceptually, diseases are the outcomes of susceptible hosts interacting with causative agents and the environment^9^. Some infectious diseases are polymicrobial, requiring the cooperation of several pathogens such as in black band disease in corals that involves primary sulfate-oxidizing cyanobacteria *Roseofilum reptotaenium* and secondary sulfate-reducing *Desulfovibrio* bacteria^10^. Confirming causation in marine invertebrate diseases can be challenging because of limited knowledge of host physiology and anatomy and difficulty in laboratory manipulation of agents associated with disease. For instance, many marine microbes cannot be cultured^3^. Against these backdrops, integrating multiple diagnostic methods such as molecular techniques paired with classic approaches (e.g., field survey, microscopy, transmission experiments) seems the best practice for disease exploration in wild marine organisms^5^.

Echinoderms are fundamental constituents structuring benthic systems, because they are ubiquitous predators/grazers, as well as prey for top carnivores affecting top-down and bottom-up regulation of communities^11–13^. A number of diseases sometimes associated with bacteria, fungi, protozoans, algae, metazoans, viruses, or often multifactorial unknown etiologies have been reported to affect this phylum sometimes leading to mass mortalities^14–20^. In some cases, diseases of echinoderms have influenced ecosystem scale processes. Examples include coral declines and algal overgrowth subsequent to urchin die offs in the Caribbean^6^ and reforestation of kelp subsequent to protozoan induced die offs of urchins in Nova Scotia^21^. The most recent echinoderm epizootic was the devastating 2013–2014 Sea Star Wasting Disease (SSWD) that killed millions of sea stars of +20 different species over an extensive geographic range of the North Pacific American Coast. Affected animals were seen often after warm seawater episodes manifesting behavioural changes, twisted arms, deflated appearance, and white lesions on the aboral dermis that rapidly progressed to tissue degradation, loss of turgor, arm loss, and death by eventual disintegration – “melting”^19,22,23^. Experimental evidence (*sensu*^24^) pointed a Parvoviridae virus, named SSaDV – sea star-associated densovirus– as the best candidate causative agent, yet this was only consistent for one asteroid species^19^. Indeed, SSWD etiology encompasses diverse cocktails of potential pathogens, and is heterogeneous across environmental conditions, geographies and species^25–27^. As a result, SSWD was recently re-named as Asteroid Idiopathic Wasting Syndrome (AIWS)^26^. The grotesque manner in which sea stars melted, together with the virulence and magnitude of this syndrome, has led to scientific concern, in addition to seizing public attention^23^. Historically though, most disease outbreaks affecting sea stars have been brief and localized and not as extensively investigated as the AIWS^8^.

Deception Island (South Shetland Archipelago) is a horseshoe-shaped island near the Antarctic Peninsula that encloses an active flooded volcano (Port Foster’s Bay; Fig. S1), subject to intense temperature fluctuations^28–30^. After the last eruption in 1970, the local benthos experienced remarkable recolonization of primarily algae and echinoderms, together with detritivore communities^31,32^. Currently, three species of echinoderms predominate: the echinoid *Sterechinus neumayeri*, the ophiuroid *Ophionotus victoriae*, and among the three most common asteroids stands out *Odontaster validus*^33,34^. The latter species is a keystone predator that has a circum-Antarctic and eurybathic range (down to 1000 m depth)^35,36^. During routine surveys in the austral summer of 2012–2013 and 2016, *O. validus* specimens were observed manifesting focal epidermal depigmentation, anorexia, and arm loss, suggesting an incipient epizootic^5,19,37^. Interestingly, the outbreak concurred with unprecedented geothermal anomalies, sudden episodic elevations in seawater temperature, and seismic reactivation of the volcano^29^. This paper describes the gross, and microscopic pathology of this disease and the role of the associated microbiota.

## Material and Methods

### Ethical statement

*Odontaster validus*, Koehler 1906, were collected and handled in agreement with all applicable international and national guidelines and regulations for the care and use of animals in accordance with the current laws of Spain and the Comité Polar Español (CPE) through the Antarctic Treaty and the Madrid Protocol on Environmental Protection to the Antarctic Treaty (‘Act on Antarctic Activities and Protection of Antarctic Environment’). The target species is not endangered or protected, and those that survived after the study were released.

### Study site and disease incidence

**Figure 1:**
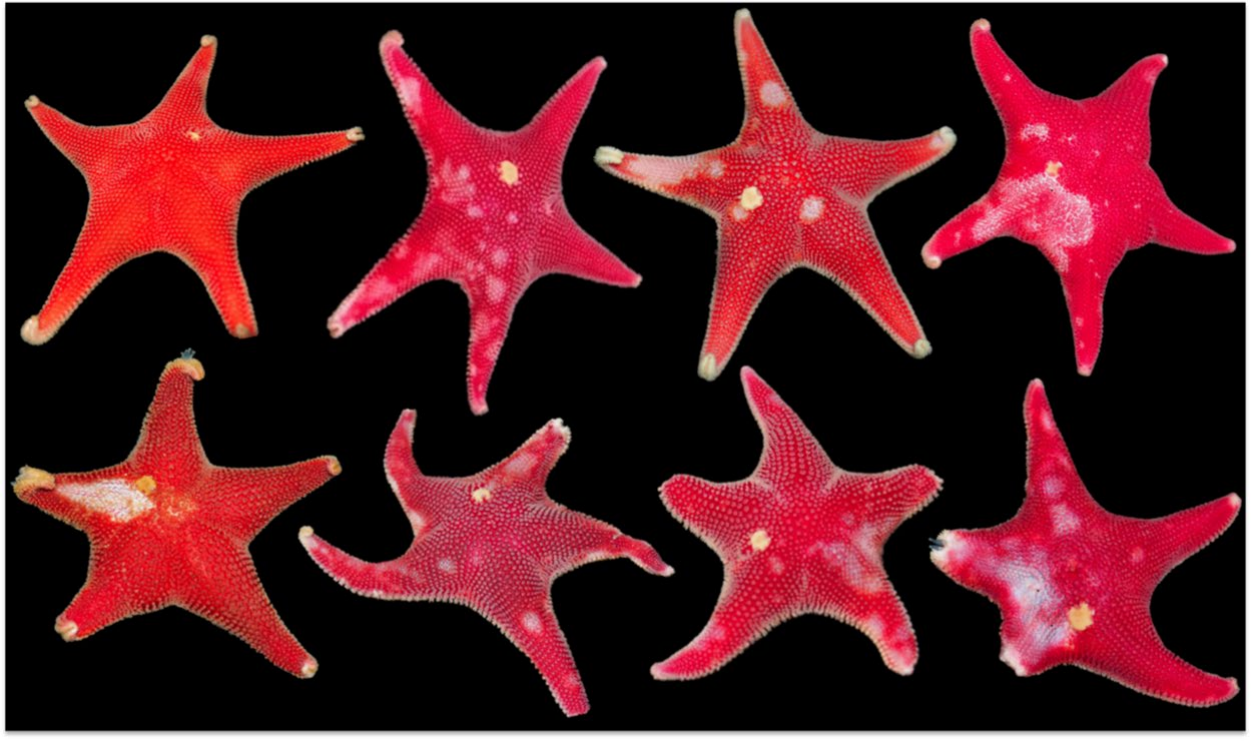
Antarctic sea star *Odontaster validus*: Normal (upper left) and diseased (remainder) specimens. Note epidermal lesions characterized by multifocal to locally extensive punctate to amorphous distinct to indistinct areas of discoloration on the dorsum. Stars on bottom row manifest partial loss of arms.

**Figure 2:**
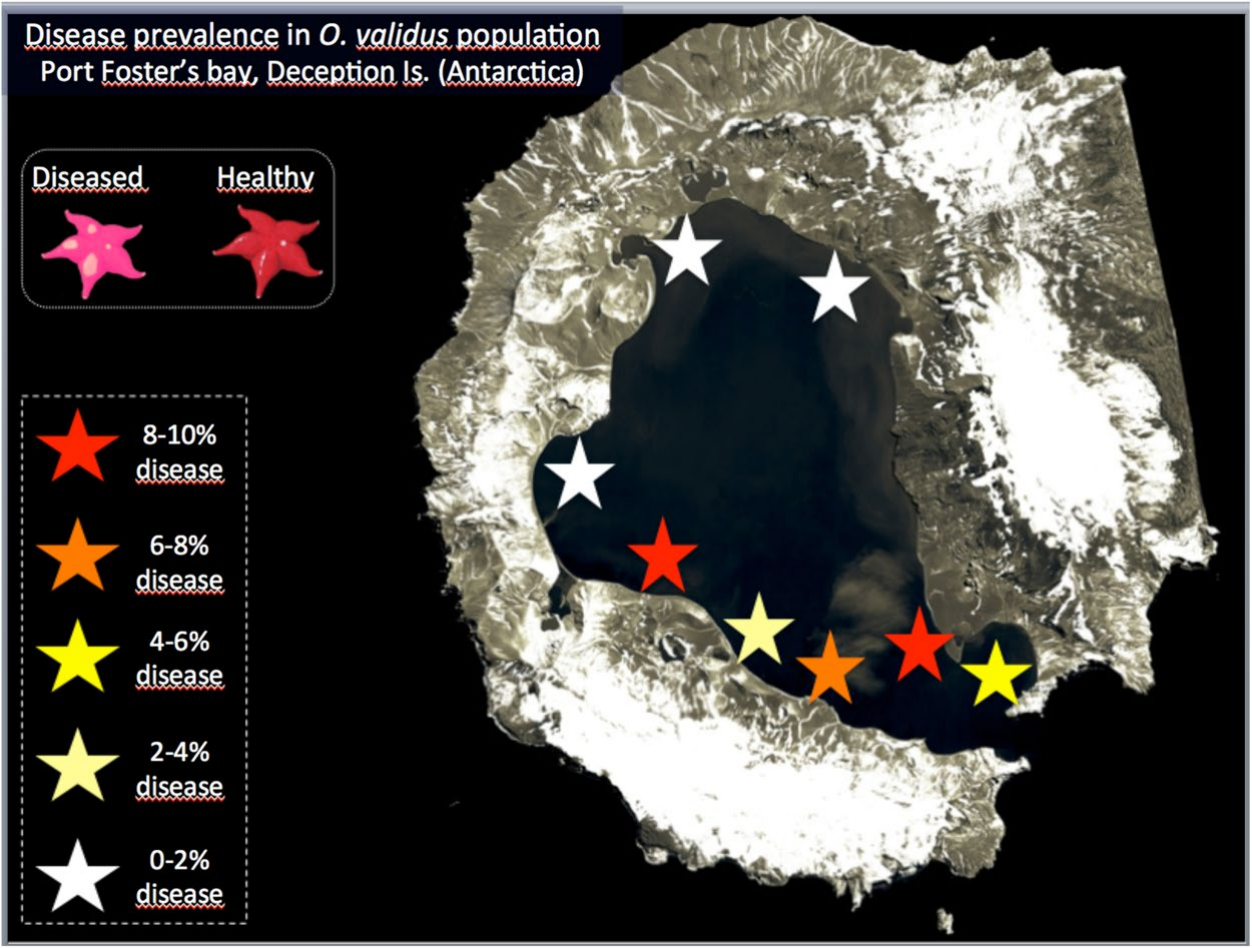
Prevalence of *Odontaster validus* sea stars affected by visually evident lesions in populations within Port Foster’s bay, Deception Island, South Shetland Archipelago (Antarctica).

Transect surveys were conducted to assess the prevalence of epidermal lesions in sea stars around Port Foster’s bay (Deception Island; Fig. S1) during the Antarctic expedition ACTIQUIM-4 (January–February 2013; Figs 1 and 2). Five haphazardly chosen replicate 50-m linear transects were surveyed at 5 m and 15 m depth at eight sites around the bay (80 transects in total; Fig. 1). Apparently healthy *O. validus* and specimens with lesions were recorded within 2 m of each transect line. The census was repeated in 2016.

### Sampling collections

Twenty-five healthy and diseased *O validus* (n = 50) were collected by SCUBA diving on February 2013. Tissue biopsies (1 cm^3^) were removed with sterile scalpel from 30 individuals (15 healthy and 15 diseased) as follows: 15 sections from healthy specimens (hereafter called “Healthy_#”), 15 affected lesion fronts from diseased sea stars (“Affected_#”), and 15 tissue areas several cm away from the lesions of the same diseased specimens (“NON_Affected_#”). Samples were divided in two subsamples, one preserved in 100% ethanol at −20 °C for DNA extraction and microbial characterization, and the other fixed in 2.5% paraformaldehyde in filtered sea water at 4 °C for histopathology studies.

### Transmission trials

To assess potential disease transmission, ten 2L-aquaria, each with one apparently healthy and one diseased sea star, were maintained in the wet laboratory for five weeks under natural sunlight. Seawater in the aquaria was pumped directly from the bay nearby the BAE (Spanish Antarctic Base) and kept at −1 °C. The seawater was completely (100%) renewed twice daily. Progression of lesions in diseased specimens and appearance of lesions in healthy individuals was monitored daily with a stereomicroscope.

### Histopathology examinations

Samples in 2.5% glutaraldehyde were embedded in paraffin at 60 °C overnight, sectioned at 5 µm, stained with hematoxylin and eosin, and examined under light microscopy.

### DNA extraction and amplification of bacterial and fungal rRNA gene

DNA from healthy and lesioned tissues of healthy and diseased stars were extracted using a modified C-TAB organic extraction protocol for amplicon deep sequencing of ribosomal gene target markers on MiSeq (Illumina), for bacterial/archaeal and fungal community composition, which were performed with a two-PCR protocol and two dual-index strategy^38,39^. In the first PCR, we used bacterial specific primers to amplify the V_3_–V_4_ region (*Escherichia coli* position: 341–785) of the small-subunit ribosomal RNA (16S) gene (341 F and 785 R)^39,40^; and fungi-specific primers ITS1F^41^ and ITS2R^42^ targeting the internal transcribed spacer 1 (ITS1) region of fungi. Amplifications were performed in 25 µl reactions with NEBNext^®^ Q5^®^ Hot Start HiFi PCR Master Mix (New England Biolabs, Inc.), 0.8 µl BSA (Bovine Serum Albumin; 20 mg/ml), 1 µl of each 5 µM primer, and 1.5 µl of template. Reactions were under the thermocycling profile: 98 °C for 2 min, then 28 cycles of 98 °C for 15 s, 53 °C for 30 s, 72 °C for 30 s, final extension at 72 °C for 2 min. The second Index PCR to attach dual indexes and Illumina sequencing adapters used forward primers with the 5′-3′ Illumina i5 adapter (AATGATACGGCGACCACCGAGATCTACAC), an 8–10 bp barcode and a primer pad; and reverse fusion primers with 5′-3′ Illumina i7 adapter (CAAGCAGAAGACGGCATACGAGAT), an 8–10 bp barcode, a primer pad. Reactions were made in 25 μl with 0.5 µl of each 5 µM primer, and 1 µl of corresponding products from first amplicon PCR reactions diluted (1:30), and with a temperature regime of: 98 °C for 2 min, then 28 cycles of 98 °C for 15 s, 55 °C for 30 s, 72 °C for 30 s, final extension at 72 °C for 2 min. The PCR products were purified and pooled equimolar on Just-a-Plate^™^ 96 PCR Purification and Normalization Kit plates following manufacturer’s instructions (Charm Biotec). Then, paired-end sequencing was performed on an Illumina MiSeq sequencer 2 × 300 flow cell at 10 pM at Core Lab, Hawai’i Institute of Marine Biology (Hawai’i, USA).

### Sequence processing and statistical analyses

Sequences demultiplexed by sample-specific barcodes were clustered/parsed using Pear 0.9.8 and quality controlled and filtered with Fastx-Toolkit. Sequence reads were then processed using the quantitative insights into microbial ecology (QIIME) pipeline 1.9.1^43^. Applying Edgar^44–46^ pipelines, low quality reads and chimeric sequences were discarded (USEARCH, UCHIME2), and OTUs (operational taxonomic units) were identified (method: UCLUST, threshold: 97%). Representative sequences were chosen by consensus and assigned a taxonomic identity (method: UCLUST) based on reference databases: Silva_123 (release version SSU/LSU 123) for Bacteria^47^ and UNITE for Fungi^48^. Prior to downstream analysis, sequences classified as “unknown” at the kingdom level, potential host co-amplification (chloroplast, mitochondrion), and contaminants were removed.

Alpha diversity (within sample) was estimated with several methods (e.g., Simpson, Fisher) and plotted using non-parametric Shannon *H’* and Chao^49^ indices, whereas OTU tables were built in Microsoft Excel with output files from QIIME and R pipelines. Beta diversity (between sample) was assessed by permutational multivariate analysis of variance – PERMANOVA^50^ at 9999 permutations to test for statistical differences (*P*-values) in bacterial and fungal community assemblages among tissue health states (i.e., Healthy_#”, “Non_Affected_#”, and “Affected_#”). Post hoc pair-wise comparisons among health states were conducted following main effects results. Bray-Curtis distance metrics at the OTU level were used to construct unconstrained two-dimensional principal coordinate analysis (PCoA) plots to visualize differences among bacterial and fungi community assemblages^51^. The similarity percentages (SIMPER)^52^ analyses based on Bray-Curtis similarity at the OTU level was used to determine the contribution of individual bacterial and fungal taxa to the dissimilarity between groups. Analysis of variance (ANOVA) was conducted on read data at the class level and also on most contributing bacterial and fungal OTUs according to SIMPER analyses to test for differences between health states. Where ANOVA resulted in significant differences, post hoc Tukey’s honest significance texts (HSD) tests were applied to assess pairwise differences. All multivariate analyses were performed in R-Studio 1.0.136 (R version 3.3.3) with the corresponding R-packages (e.g., PhyloSeq, Vegan, Bioconductor).

### Data availability

The sequence data set was deposited in the NCBI Sequence Read Archive (SRA) database (accession numbers: SRP131970). Other related datasets generated during and/or analysed during the current study are available from the corresponding author on request.

## Results

### Disease incidence

**Figure 3:**
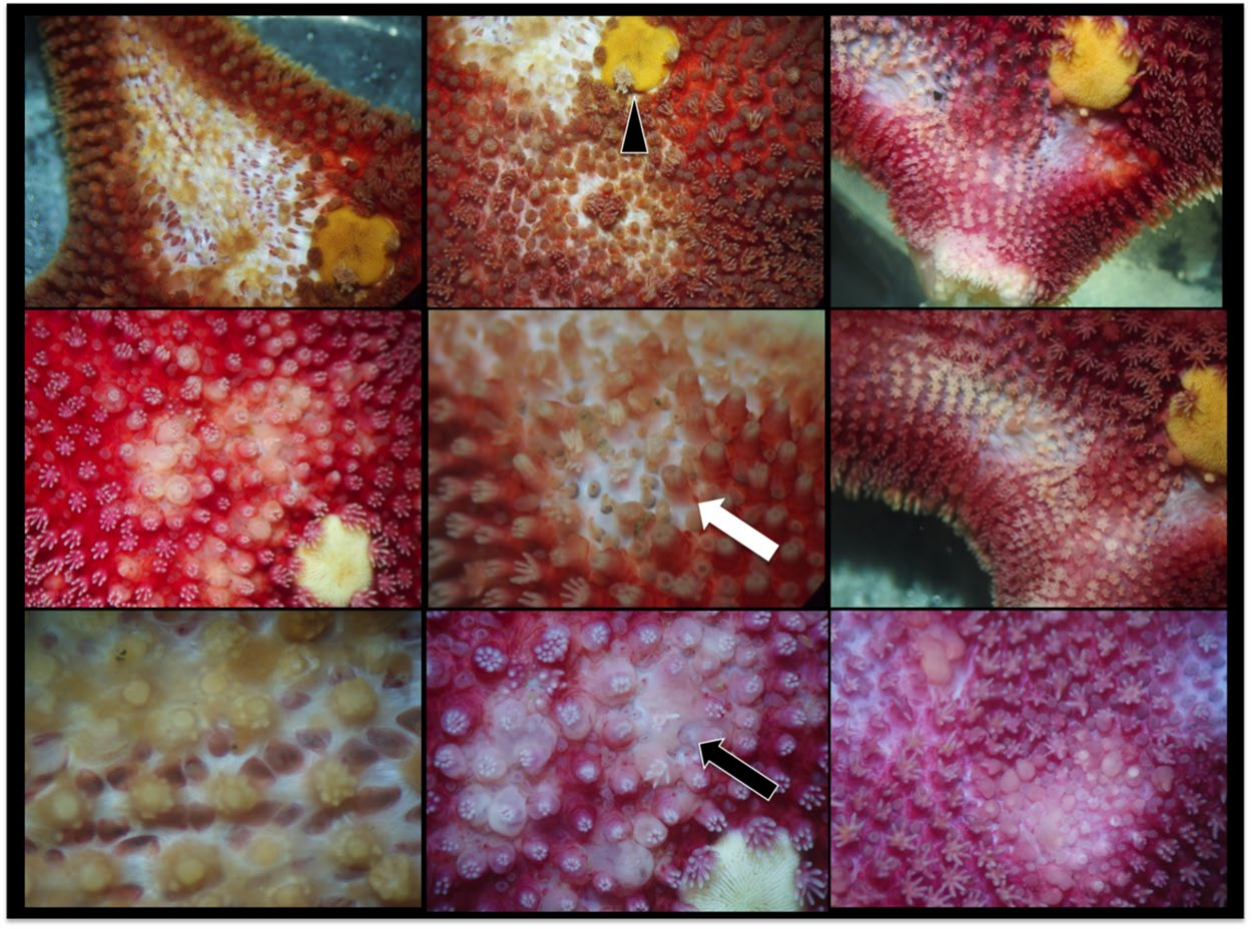
Higher magnification under stereoscope of epidermal lesions in *Odontaster validus* showing depigmentation (white arrow), and erosion of paxillae and epidermal ulceration (black arrow). Arrowhead pointing to the madreporite.

Lesions were limited to *O. validus* with a prevalence ranging from 0% at most inner areas of Port Foster’s caldera, up to 10% at sites closer to the entrance –Neptune’s Bellows (Fig. 2). Apparent early stages of disease comprised multifocal distinct to indistinct 1–5 mm pale to white spots that coalesced to larger ulcers leading to decay of spines, papulae and ablation of paxillae, with more severe cases revealing underlying ossicles or total amputation of arms (Figs 1 and 3).

### Transmission trial

In aquaria, epidermal lesions in affected stars progressed almost imperceptibly (1–2 mm increase in size over 5 weeks’ observation). Animals going from asymptomatic to the severe signs of disease were not observed in the time lapse during our campaigns. We saw no evidence of transmission between lesion and healthy stars.

### Histopathology

**Figure 4:**
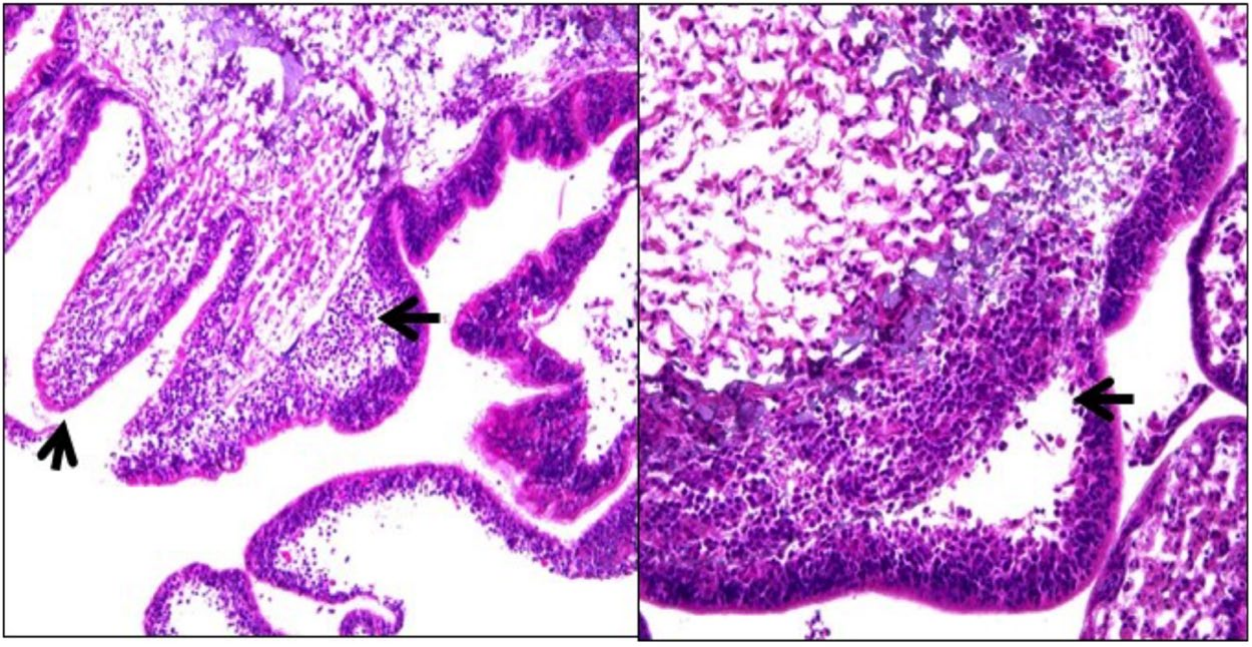
Histological sections of *Odontaster validus* epidermal lesions: (left) note inflammatory cell infiltrate lifting off epidermis from underlying ossicle (big black arrow) with diffuse ulceration (small black arrow); (right): note marked subepithelial infiltrates of inflammatory cells with cleft formation (arrow).

Histology of gross lesion fronts of diseased animals revealed cleft formation between epidermis and dermis with spaces infiltrated by hemocytes (inflammation). In more severe lesions, there was full thickness epidermal necrosis and ulceration with associated hemocyte infiltrates lifting off the epidermis from the underlying ossicles (ulceration). No characteristic accumulations of microorganisms were seen associated with the lesion. Five of 15 tissues with lesions had histologic evidence of inflammation/ulceration, whereas no histopathological signs were found in the 15 non-lesion tissues examined (Fig. 4).

### 16S rRNA gene bacterial diversity and community composition

**Figure 5:**
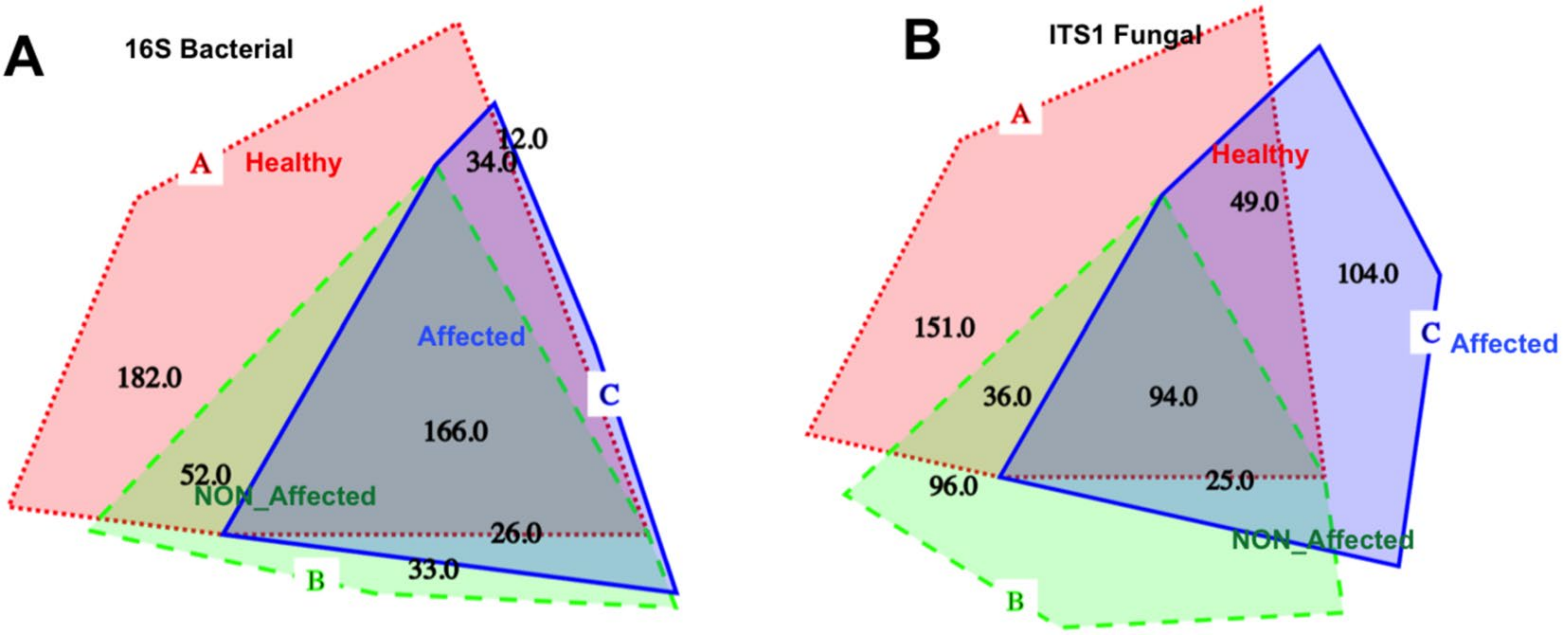
Proportional Venn Diagrams depicting represented OTUs by tissue type for the bacterial (**A**) and fungal (**B**) communities associated with *Odontaster validus* sea stars. Healthy (in red): tissues from healthy sea stars; NON_Affected (in green): apparently healthy tissues from diseased specimens; and Affected (in blue): diseased tissues from diseased specimens.

Sequences retrieved for the bacterial 16S hypervariable V_3_–V_4_ region yielded, after quality control and chimera filtering, 189,046 reads with an average length of ~427 bp. According to the number of observed OTUs, Shannon and Fisher indexes, and Chao1 estimation of species richness based on normalized reads (n = 1545), the bacterial diversity of tissues from healthy sea stars was remarkably higher than that from diseased samples, including apparently non-affected sections and lesion fronts (Table 1, Fig. S2). Simpson diversity index was similar for healthy tissues from healthy and affected sea stars, but yielded lower values in lesion areas. A total of 505 OTUs were detected based on a sequence similarity criterion of 97%, of which 434 were associated with Healthy tissues, whereas 277 and 238 were found in NON_Affected and Affected sections, respectively. In concordance with diversity statistics, the number of unique OTUs was the highest in healthy sea stars (182), with respect to apparently healthy (33), and lesion sections (12) from the diseased specimens (Table 1; Fig. 5).

**Table 1:** Summary of 16S rRNA bacterial and ITS-1 fungal communities profiling from healthy and diseased *Odontaster validus* sea stars.

Status	Healthy seastar		Diseased seastar				TOTAL	
Tissue	Healthy		NON_Affected		Affected			
Replicates (n)	15		15		15		45	
Genetic marker	**16S**	**ITS-1**	**16S**	**ITS1**	**16S**	**ITS-1**	**16S**	**ITS1**
Reads per sample	1,545–6,271	3,129–3,4871	2,975–6,890	1,378–2,3487	2,269–5,917	1,380–1,5095	189,046	335,754
Average length							427	272
Fisher index	63.25436	39.62805	36.64054	31.09836	31.64202	35.7209	63.08028	64.90119
Simpson index	0.2812992	0.9484165	0.2953405	0.9442799	0.19308	0.9325904	0.2601939	0.9522523
Observed OTUs^a^	434	330	277	251	238	272	505	555
Unique OTUs^b^	182	151	33	96	12	104	227	351
Number of Phyla	13	3	9	3	10	4	14	4
Number of Classes	26	16	17	17	17	14	28	18
Number of Order	52	33	32	33	32	31	57	40
Number of Families	100	47	61	40	54	46	106	63
Number of Genera	182	90	95	79	79	82	207	136

Abbreviations: OTUs, operational taxonomic units; ITS, interspatial tanscripted spacer.

Alpha diversity statistics were calculated with phyloseq R-package on RStudio.

^a^OTUs can be shared among multiple samples and are based on 97% sequence similarity criteria according to Silva_123 database.

^b^Number of unique OTUs characteristic for each tissue type after the sequences were referenced and combined by QIIME.

**Figure 6:**
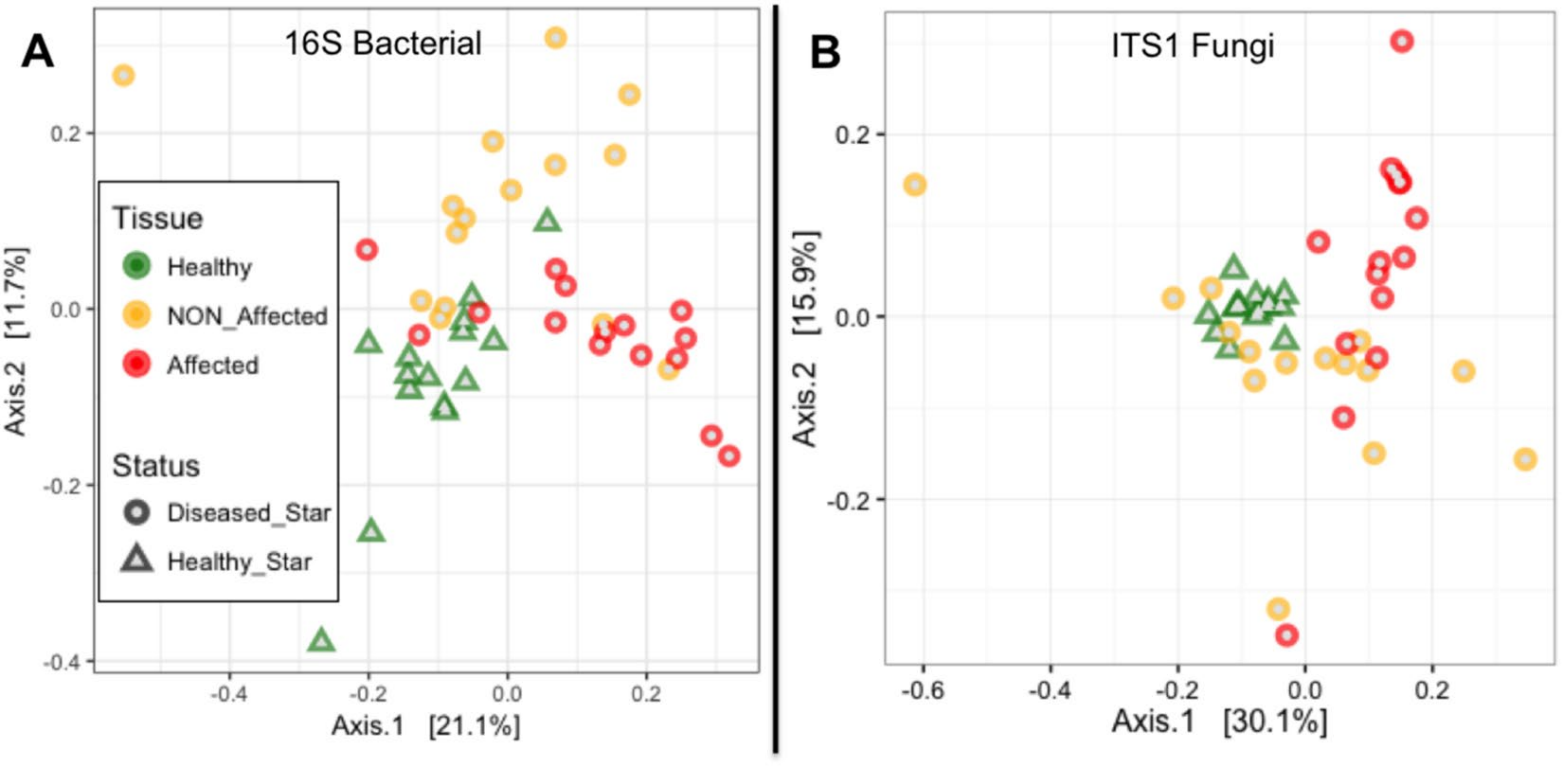
Principal Components Analysis (PCoA) plots based on Bray-Curtis distances of *Odontaster validus* associated bacterial (**A**) and fungal (**B**) communities composition across three tissue types: healthy tissues from healthy sea stars (Healthy; green triangles), apparently healthy tissues from diseased sea stars (NON_Affected; yellow circles), and affected tissues from diseased sea stars (Affected; red circles). The proximity of samples on the PCoA illustrates the similarity of bacterial or fungal communities.

At the species level (97% sequence similarity), bacterial communities associated with *O. validus* were significantly different between health states (df = 1, pseudo-F = 1.996, *P*_*perm*_ < 0.05) and among tissue types (df = 1, pseudo-F = 2.923, *P*_*perm*_ < 0.001). For the same health status, specimen replicates displayed similar bacterial communities demonstrating homogeneity within tissue types (df = 28, pseudo-F = 1.318, *P*_*perm*_ > 0.05). Healthy and Affected tissues exhibited significant difference (df = 1, pseudo-F = 5.486, *P*_*perm*_ < 0.01), and in diseased sea stars the bacterial composition differed between Affected and NON_Affected tissues (df = 1, pseudo-F = 4.874, *P*_*perm*_ < 0.01). No significant differences were detected between healthy tissues sampled from healthy specimens versus healthy tissues sampled from diseased sea stars away from the lesion areas (i.e., Healthy and NON_Affected; df = 1, pseudo-F = 0.872, *P*_*perm*_ > 0.05; see Table S1). Segregation of bacterial community compositions associated with tissues of different health status can be visualized in PCoA ordination, where Affected tissues clustered loosely towards the positive end of the first axis. Along the negative end of this axis 1 there was a transition towards a loose clustering of Healthy and NON_Affected tissues, which shifted from one another along the centre of axis 2 (Fig. 6).

**Figure 7:**
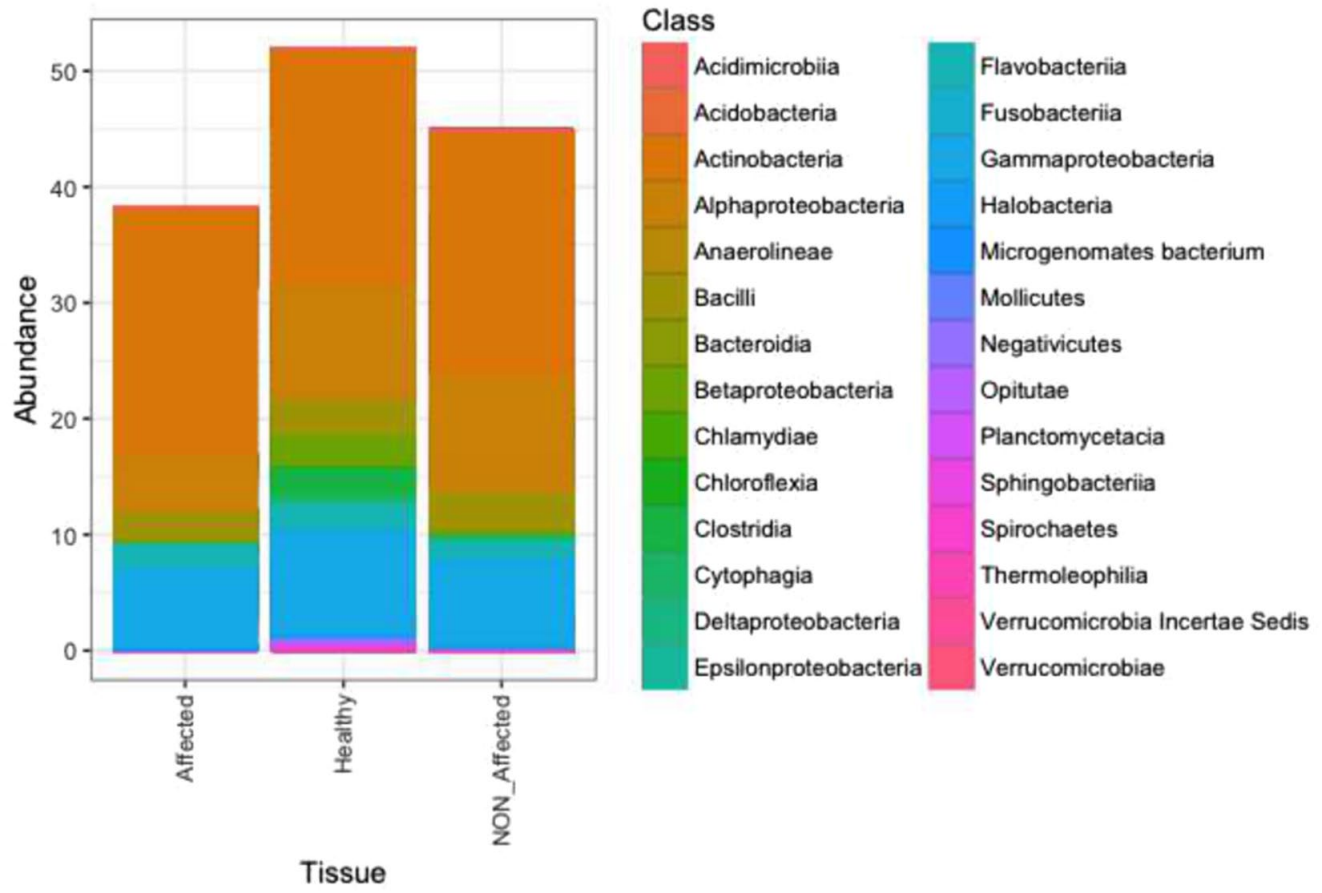
Class-level taxonomic diversity profiling of bacterial communities associated with tissues from healthy sea stars (Healthy), with apparently healthy (NON_Affected) and diseased (Affected) tissues from diseased specimens of *Odontaster validus*. “NA”: non-determined taxa.

The most represented phylum in all tissue types was Actinobacteria (Fig. 7), which was dominated by the genus *Rhodococcus* (98%), and in particular by B_OTU240 (78.25% in Affected *versus* 63.16% in Healthy and 55.18% in NON_Affected, SIMPER, *P*_*ANOVA*_ < 0.05). According to SIMPER, *Rhodococcus* B_OTU240 along with B_OTU338 (*Acinetobacter* sp.) and B_OTU4 (*Moritella* sp.) are the most influencing OTUs associated with diseased sea stars (Table S2 and Fig. S3). Major differences among healthy states, though, were due to variations in the relative abundance of OTUs affiliated with members of phyla Proteobacteria, Firmicutes, and Bacteroidetes. These were consistently more highly represented in Healthy *O. validus*, followed by NON_Affected, and least abundant in lesion fronts. At the class level, graphing data and statistics (*P*_*ANOVA*_ < 0.05) showed that Alphaproteobacteria, Bacilli, and Cytophagia were particularly scant in lesion fronts. While Betaproteobacteria, Clostridia, Gammaproteobacteria, Sphingobacteriia, Negativicutes, and Bacteroidia were more abundant in Healthy sections, they were less abundant in both, NON_Affected and Affected tissues from diseased *O. validus* (Fig. 7). Twenty-one OTUs revealed >0.1% contribution to dissimilarity (SIMPER) and yielded loose clustering in PCoA plotting between health states (Fig. S3; see Table S2 and ESM1 for more detailed information).

### ITS1 rRNA gene fungal diversity and community composition

Fungi associated with sea star tissues resulted in 335,754 classifiable, non-chimeric reads, averaging *ca*. 272 bp. The diversity of fungal communities from healthy sea stars was higher than those from Affected and NON_Affected tissues of diseased individuals, as indicated estimations of observed OTUs, Shannon and Fisher indexes, and Chao1 (normalized reads n = 1378; Table 1, Fig. S2). Simpson diversity index showed equivalent results for tissues from all health states. The total number of OTUs at a sequence similarity of 97% was 555. Healthy tissues yielded 151 unique OTUs, 104 were found for disease lesion fronts and 96 for apparently healthy sections from diseased specimens (Table 1; Fig. 5).

The PERMANOVA analysis did not reveal significant differences at the species level (97% sequence similarity) in the overall fungal composition associated with individual health states (df = 1, pseudo-F = 1.04, *P*_*perm*_ > 0.05), nor among different tissue types (df = 1, pseudo-F = 0.972, *P*_*perm*_ > 0.05). For the same health status, specimen replicates displayed homogeneity of fungal communities within tissue types (df = 28, pseudo-F = 1.005, *P*_*perm*_ > 0.05; Table S1). Although there was no statistical difference among health states at the community level, ordination plotting and SIMPER integrated with ANOVA analyses showed the existence of certain explanatory fungal taxa. In the PCoA ordination, Healthy-associated fungal communities appear tightly clustered and separated along axis 1 from a loose clustering corresponding to disease lesion fronts, which dispersed along the second PCoA axis. Fungi populations of NON_Affected tissues from diseased sea stars were the most scattered along both axes (Fig. 6).

**Figure 8:**
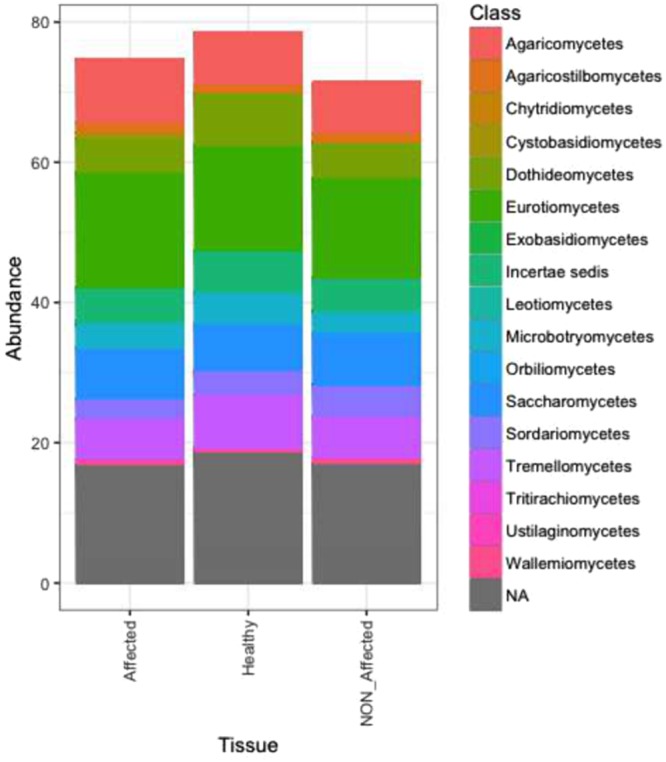
Class-level taxonomic diversity profiling of fungal (**B**) communities associated with tissues from healthy sea stars (Healthy), with apparently healthy (NON_Affected) and diseased (Affected) tissues from diseased specimens of *Odontaster validus*. “NA”: non-determined taxa.

No significant differences in fungal communities were recorded in samples coming from healthy versus diseased sea stars. Ascomycota followed by Basidiomycota were predominant in the three tissue types, along with a very small representation of Chytridiomycota. A notable percentage of sequences were unassigned –Fungi sp. Qualitative variability based on graphical and/or statistical (*P*_*ANOVA*_ < 0.05) data was observed for some class groups. Dothideomycetes, Tremellomycetes, and Microbotryomycetes were more abundant in Healthy, than in NON-Affected of Affeted tissues, whereas Agaricomycetes, Wallemiomycetes, Cystobasidiomycetes, and Eurotiomycetes were more associated with lesion fronts (Fig. 8). Qualitative changes became more evident at the OTU level. Indeed, from the SIMPER analysis, 57 OTUs contributed >0.3% in discriminating among health states. These provided relatively tight clusters (PCoA) according to tissue types (Fig. S3; see Table S3 and ESM1 for more detailed information).

## Discussion

This is the first description of a novel emerging echinoderm disease occurring in the Antarctic Ocean based on a combination of microbial community profiling and pathology. Sea stars are abundant and comprise various species at Deception Island^33,34^; however, only one species, the most abundant – *O. validus–* manifested lesions. The outbreak was first detected in the austral summer of 2012–2013, coinciding with seawater and soil fluctuations characterized by spikes in elevated temperature, and increase in the frequency of seismic events. Similar geothermal anomalies were not recorded in the preceding or following years^29^. Prevalence of skin lesions in asteroids within Port Foster Bay ranged from barely over 0% in the innermost sites to 10% at locations adjacent to the bay’s opening, broadly following the gradient of species diversity in the region^33,34^. This trend was consistent over two surveys (2013,2016), and was correlated with *O. validus* distribution, which is largely driven by colonization, sedimentation, and sea current regimes^34^. In this context, what we saw in *O. validus* was similar to other disorders affecting echinoderms where disease was more prevalent after periods when temperature peaks, and in locations with higher agglomeration of counterparts, suggesting perhaps a density dependent process (e.g.,^15,23,26,27^). Cannibalism seems to be a rare behaviour in *O. validus*, hence density-dependent intraspecific aggression is an unlikely explanation for the lesions^53^. Physical or chemical traumas would neither explain the lesions; on the one hand because the closed shape of the bay reduces the entrance of strong currents or icebergs to cause abrasions; and on the other because human presence is too limited to generate pollution and chemical stress. In all these cases such insults would presumably be found at constant rates every year, yet, we only saw lesions in certain years.

The clinical signs described here differed from the AIWS of the NE Pacific, where multiple (>20) species were affected manifesting much more severe and rapidly progressing apparently transmissible clinical signs, i.e., wide spread arm loss and complete body disintegration^19,23,54^. Susceptibility of individuals to AIWS differed according to age (lower and slower in juveniles compared to adults), subhabitat, and sea star colour^22,23^. Moreover, subsequent to the AIWS epizootic, there was a sharp reduction in predation rate^23^. In contrast, in our study, only a single species was affected, and clinical signs were relatively mild (multifocal ulceration), did not appear to progress rapidly, and did not seem transmissible. This might be explained in part by the slower metabolism of Antarctic organisms in contrast to organisms in more temperate climates^55,56^, so it seems plausible that disease transmission and progression could be slow and, thence, imperceptible during the duration of a regular scientific polar campaign (~1.5 months). Another explanation could be that the etiology of lesions in *O. validus* is completely different than that affecting animals in AIWS. Colouration in *O. validus* can vary with age with juveniles being pink, purple, to orange and adults being bright red^36^. Most *O. validus* affected exhibiting lesions were red adults; however, juveniles of other colour-morphs were uncommon, so age comparisons were not possible. Additionally, behavioural consequences of the disease such as inactivity and appetite loss were seldom observed in predation experiments conducted in 2012–2015 (e.g.,^57^), This may be partially explained by stress, and the loss of respiratory surface and papulae.

Whilst microscopy revealed a moderate to marked inflammatory response with epidermal ulceration, there was no evidence of infectious agents visible on light microscopy, thus, it is unlikely that organisms visible at that level such as fungi, bacteria^16^ or parasites^58^ were responsible for the epidermal lesions seen here. Moreover, clinical signs (lack of progression, transmissibility) also argued against an infectious cause. However, light microscopy has low to no resolution when visualizing microorganisms of the size of mycoplasma and viruses, so these remain as possible infectious agents. Alternatively, non-infectious causes should also be considered, as skin ulceration is a common response to a variety of insults in echinoderms^59,60^, which may or may not have an infectious etiology.

Microbial community analyses failed to reveal bacterial or fungal putative pathogens associated with lesions. The consistently lower bacterial and fungal diversity found in the lesion fronts of affected asteroids contrasts with other studies, where an increase in microbial diversity followed a disease or stress response in, corals^61,62^, sponges^63^, and also echinoderms^58^, but agrees with other findings in marine invertebrates (e.g.,^63^). Some authors suggest that diseased tissues represent favourable niches for colonization of secondary invaders inflating microbial diversities due to host’s immune disruption caused by stress, together with a higher availability of nutrients from decaying tissues^64^. In fact, one of the major constraints when exploring the etiology of marine diseases during non-initial symptomatic stages (e.g., bleaching, spotting, or rotting) is to distinguish real pathogens from saprophytes growing on decaying hosts^24,65^. In this study, the relatively low microbial diversities recorded in the diseased fronts of *O. validus* may be explained by either a lower colonization rate of opportunistic microbes under Antarctic temperatures^55^ or to antimicrobial inhibitors (as suggested in^63^) elicited by immune response of the host, as described in other asteroid syndromes^54^. It is possible that the differences in microbiota could be due to microbiome shifts prior to the manifestation of lesions, leading to upsurge of opportunistic polymicrobial infections, and development of lesions without the implication of ‘legitimate’ pathogens (*i.e*. microbial dysbiosis *sensu*^65^). The empirical data to support this could be the higher consistency in microbial communities associated with healthy states across individuals, with respect to the lesion fronts and apparently healthy skin areas from specimens of diseased sea stars. All this illustrates that absent clear evidence of pathogens associated with lesions at the tissue and cellular level, conclusions gained from molecular studies are necessarily limited.

Actinobacteria were “core” members of the associated flora of *O. validus*, in agreement with other marine microbiomes^66,67^. The bulk of sequences within this phylum belonged to *Rhodococcus*, which harbours benign steroid producers and a few pathogens^68^. Proteobacteria, followed by Firmicutes and Bacteroidetes, displayed double the sequence abundances in Healthy tissues as compared to lesion fronts. Bacteroidetes has been associated with healthy states but also with disease in contrasting studies^64,69^; whereas, Proteobacteria comprises pathogens, opportunists, and nitrogen fixators^70,71^. Three Rhodobacteraceae OTUs and one *Acinetobacter* were particularly abundant in NON_Affected sections from diseased sea stars, where they could have proliferated as pre-invasive pathogens^72,73^. Accordingly, the family Rhodobacteraceae was correlated to coral disease outbreaks^74^. Instead, *Clostridium* (Firmicutes) strains, appearing exclusively in healthy sea stars, suggest symbiotic interactions^75^.

As in other marine invertebrates, Ascomycota and Basidiomycota were the most common fungi in *O. validus*, along with a small proportion of Chytridiomycota (mostly saprobic)^76,77^. Many sequences were not classified due to limitations in fungal databases^76^. Saccharomycetes yeasts contributed to the flora of all health states, where they could be forming symbiotic matrices^77^. Certain taxa with wide metabolic ranges in Dothideomycetes, Tremellomycetes, Microbotryomycetes, and Sordariomycetes were variably correlated to healthy or NON_Affected tissues, likely driving microbial shifts prior to lesion manifestation^70,77–79^. Eurotiomycetes, Agaricomycetes, Wallemiomycetes, and Cystobasidiomycetes were more common in diseased sea stars, and probably harboured opportunistic strains proliferating in distressed host tissues^77,80^. Besides the opportunistic *Trichosporon guehoae*^81^ (F_OTU556, Eurotiales), there were three exclusive strains (F_OTU520, F_OTU165, F_OTU450) that were closely associated with lesion fronts.

Temperature plays a critical role in pathogenesis of disease in marine invertebrates and poikilothermic vertebrates^19,22,23,54,82–84^. Antarctica is one of the most susceptible areas to climate change on Earth^85^, and Deception Island is one of the most temperature variable sites in the Southern Ocean^28^. Furthermore, marine ice has been proposed as an important abiotic reservoir for pathogens^82^. After 5-years of observation of this enigmatic disorder, the incidence of disease symptoms in *O. validus* populations seem to oscillate locally between over 0% and 10%. That said, and considering the coincidence of the first outbreak in 2012–2013 with an unprecedented local thermal anomaly^29^, we need to be vigilant as the disease could potentially boost with increasing temperatures. The isolated and unexpected nature of this disease illustrates gaps in our knowledge of demographics and health of marine invertebrates that play an inordinately important role in Antarctic benthic ecology. Most Antarctic species are characterized by a relatively slow growth and maturation times^56^. If this disease decreases sea stars’ populations in the coming years, recovery could be slow^23,86–88^, with direct consequences to the ecosystem^35,36^. Future studies might focus on clarifying the fitness and demographic impacts of epidermal lesions on *O. validus* and developing tools to understand pathology and pathogenesis, correlated with year-round temperature data. In the face of the alarming mortalities affecting sea star populations in the past years (e.g.,^19,22,23^), we believe this and upcoming research involving disorders in keystone Antarctic species are extremely relevant and urgent.

## Electronic supplementary material

Figures S1-S3

ESM1

Table S1

Table S2

Table S3
