# Author Correction: Exploring the pathology of an epidermal disease affecting a circum-Antarctic sea star

**DOI:** 10.1038/s41598-018-31112-2

**Published:** 2018-08-21

**Authors:** Laura Núñez-Pons, Thierry M. Work, Carlos Angulo-Preckler, Juan Moles, Conxita Avila

**Affiliations:** 1 Section Biology and Evolution of Marine Organisms (BEOM), Stazione Zoologica Anton Dohrn (SZN), Villa Comunale, 80121 Napoli, Italy; 2 Smithsonian Tropical Research Institute (STRI), Tupper/Naos/Bocas del Toro Labs, Ancón, 0843-03092 Panamá City, Republic of Panama; 3 US Geological Survey, National Wildlife Health Center, Honolulu Field Station, Honolulu, HI 96850 USA; 4 Department of Evolutionary Biology Ecology and Environmental Sciences, and Biodiversity Research Institute (IrBIO), University of Barcelona, Faculty of Biology, Av. Diagonal 643, 08028 Barcelona, Catalonia Spain; 5 Museum of Comparative Zoology & Department of Organismic and Evolutionary Biology, Harvard University, 26 Oxford Street, Cambridge, MA 02138 USA

Correction to: *Scientific Reports* 10.1038/s41598-018-29684-0, published online 27 July 2018

In the original version of this Article, the ORCID IDs for Laura Núñez-Pons, Thierry M. Work and Conxita Avila were omitted.

This error has now been corrected in the HTML and PDF versions of the Article.

