# Supplementary material for: Exploring the pathology of an epidermal disease affecting a circum-Antarctic sea star
Source: Sci Rep. 2018 Jul 27;8:11353. doi: 10.1038/s41598-018-29684-0 (PMC6063859; doi:10.1038/s41598-018-29684-0)
Supplement: Supplementary file 1 — Figures S1-S3 [file 41598_2018_29684_MOESM1_ESM.pdf]

Exploring the pathology of an epidermal disease affecting a circum-Antarctic sea star

Laura Núñez-Pons[1,2*], Thierry M. Work[3], Carlos Angulo-Preckler[4], Juan Moles[5], Conxita Avila[4]

Supplementary Figures:

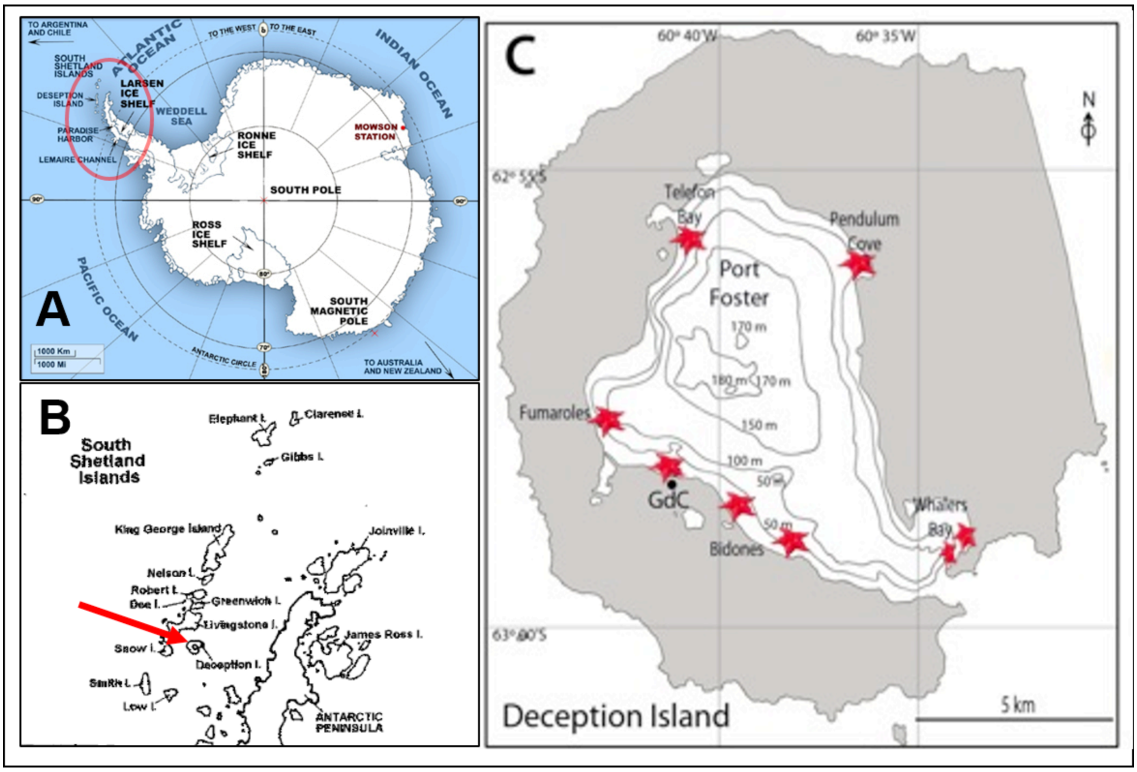

Figure S1 Location of the study: Antarctic continent (**A**); South Shetland Archipelago (**B**); and Deception Island with the eight sampling stations where 50 m linear transects were surveyed to estimate the prevalence of the lesions in sea stars (**C**). GdC: Gabriel de Castilla (Spanish Antarctic base).

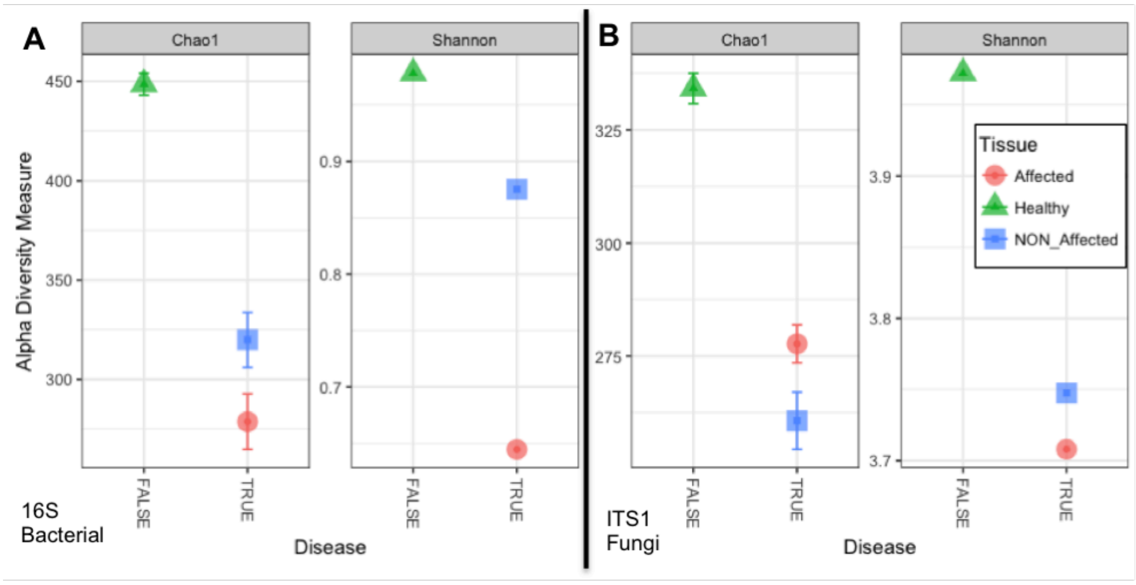

Fig. S2 Alpha diversity for the associated (**A**) bacterial and (**B**) fungal communities in tissues from healthy sea stars (Healthy; Green triangle), and in apparently healthy (NON_Affected; blue square) and affected (Affected; pink-red circle) tissues from diseased specimens of *Odontaster validus*, expressed as the average Chao1 and Shannon indexes (means ± s.e.).

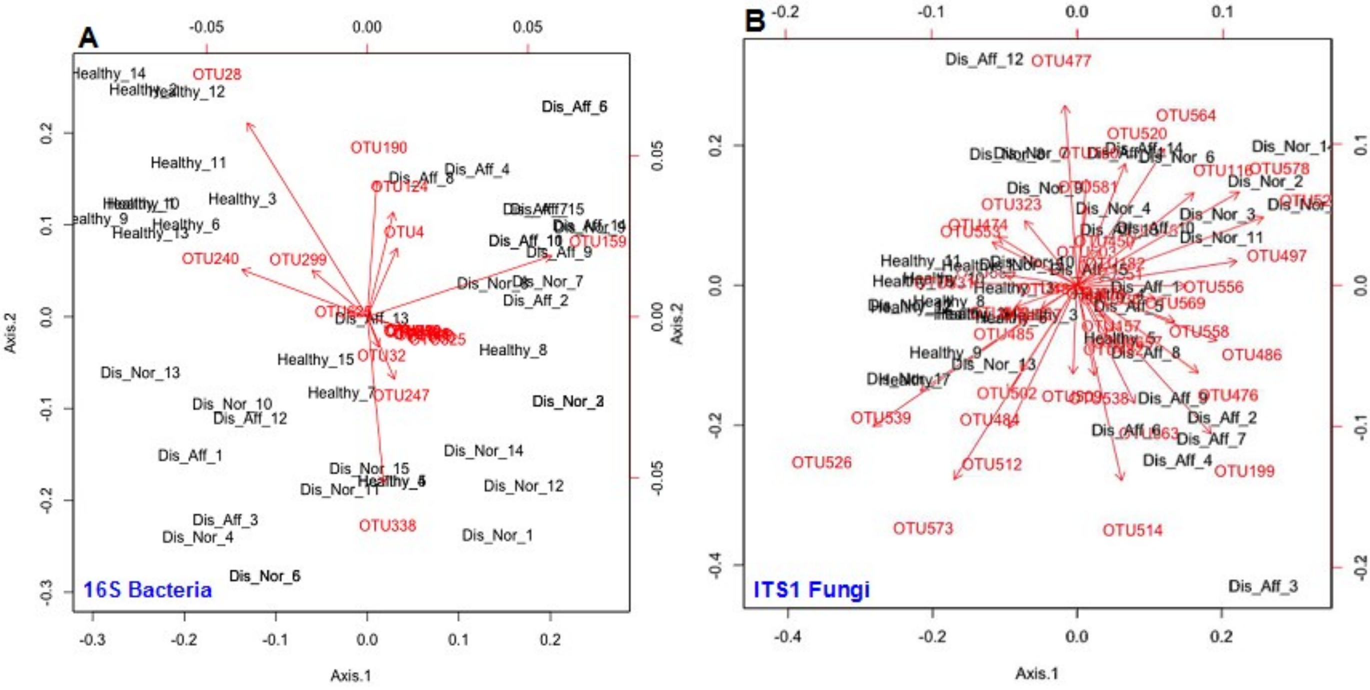

Figure S3. Principal Component Analysis (PCoA) plot based on similarities of (**A**) bacterial and (**B**) fungal assemblages. Variables are the most descriptive OTUs among health states according SIMPER analysis (Tables S1 and S2) for Bacteria –B_OTUs#, and Fungi –F_OTUs# (> 0.3% contribution to dissimilarity). "Healthy_#" were samples from healthy *O. validus*; "Dis_Nor_#" were apparently healthy tissues from diseased sea stars; and "Dis_Aff_#" were sections from the diseased lesion fronts. The vectors in red indicate the OTUs that most contribute to discriminate tissue types.
