# Supplementary material for: Exploring the pathology of an epidermal disease affecting a circum-Antarctic sea star
Source: Sci Rep. 2018 Jul 27;8:11353. doi: 10.1038/s41598-018-29684-0 (PMC6063859; doi:10.1038/s41598-018-29684-0)
Supplement: Supplementary file 2 — ESM1 [file 41598_2018_29684_MOESM2_ESM.pdf]

Exploring the pathology of an epidermal disease affecting a circum-Antarctic sea star

Laura Núñez-Pons[1,2*], Thierry M. Work[3], Carlos Angulo-Preckler[4], Juan Moles[5], Conxita Avila[4]

Electronic Supplementary material:

RESULTS SECTION.

Results of the most descriptive bacterial and fungal OTUs in defining the different tissue types in *Odontaster validus* disease.

16S rRNA gene bacterial OTUs contribution.

Several of these most discriminating OTUs were exclusive to Healthy samples; e.g., B_OTU325 (*Azovibrio* sp.), B_OTU60 and B_OTU352 (*Clostridium* spp.), B_OTU309 (Comamonadaceae sp.), B_OTU188 (*Porphyromonadaceae bacterium NM7*), B_OTU194 (*Acidaminobacter* sp.), B_OTU132 (*Zoogloea sp. UNPF36*), B_OTU210 (*Pelosinus* sp.) and B_OTU14 (*Azospira* sp.). Others were largely correlated to Healthy (B_OTU299 –*Colwellia* sp.) or also NON_Affected tissues, e.g., B_OTU28 (Rhodobacteraceae sp., B_OTU625 (*Fabibacter* sp.) and B_OTU625 (*Fabibacter* sp.). B_OUT124 (*Polaribacter* sp.) was more common in lesion fronts. Finally, there were two OTUs highly descriptive for NON_Affected sections (e.g., B_OTU190 –*Roseobacter* clade NAC11-7 and B_OTU159 –*Sulfitobacter* sp.), while B_OTU338 (*Acinetobacter* sp.), B_OTU247 (*Bacillus firmus*) and B_OTU32 (*Geobacillus stearothermophilus*) were similarly contributing to all health states (Fig. S3A and Table S1).

ITS rRNA gene fungal OTUs contribution.

The most represented OTU was F_OTU573 –Eurotiales sp. (17.92% in NON_Affected, 19.46% in Healthy and 22.75% in Affected). There were OTUs more related to disease lesion

fronts, a few being exclusive (F_OTU520, F_OTU165 –*Trichosporon guehoae,* and F_OTU450), whereas others were defining (F_OTU556 –Eurotiales sp., F_OTU514 – *Rhodotorula mucilaginosa*, F_OTU199 –*Candida parapsilosis*, F_OTU486 –Polyporales sp., F_OTU157 –*Rhodosporidium babjevae*, F_OTU581 –Tremellales sp., F_OTU476 – *Penicillium multicolor,* F_OTU569 – *Microporus affinis*, F_OTU548 –*Cyberlindnera jadinii*, F_OTU551 – *Malassezia restricta*, F_OTU530 –Nectriaceae sp., F_OTU115 –*Penicillium bialowiezense*, F_OTU33 –*Cryptococcus diffluens*, F_OTU –*Penicillium glandicola*, F_OTU55, F_OTU543 – *Sistotremastrum guttuliferum*). Healthy tissue samples harboured the highest incidence of exclusive OTUs (*i.e.*, F_OTU536 – Hypocreales sp., F_OTU482 –*Phaeococcomyces nigricans*, F_OTU327 –*Cryptococcus antarcticus*, F_OTU500, F_OTU483, F_OTU485, F_OTU531 – *Periconia* sp., F_OTU571 – *Ceratobasidium* sp.); and had also defining OTUs (F_OUR539, F_OTU565 – *Scopuloides hydnoides,* F_OTU474 –*Pyrenochaetopsis leptospora*, F_OTU499 – *Cryptococcus magnus,* F_OTU566 – *Glaciozyma martini*, F_OTU568 –*Aureobasidium pullulans*, F_OTU513 –*Mrakia frigida*, F_OTU502, F_OTU549, F_OTU369 –*Polychaeton citri*). Among the OTUs mostly associated to apparently healthy NON_Affected tissues from diseased sea stars, a few were exclusive (F_OTU477 –Pleosporales sp., F_OTU160 and F_OTU116 –*Monographella cucumerina*); whereas others were defining (F_OTU564, F_OTU578 –Sordariomycetes sp., F_OTU497, F_OTU538 – *Candida sake,* F_OTU559 – *Debaryomyces nepalensis*, F_OTU528, F_OTU475 –*Trichosporon asahii,* F_OTU553, F_OTU182 –Hypocreales sp., F_OTU323 –*Devriesia lagerstroemiae*, F_OTU563, F_OTU572 –*Candida orthopsilosis*, F_OTU464 –*Aspergillus gracilis*, F_OTU19 –*Wallemia mellicola*). Certain OTUs were associated to Healthy and apparently healthy NON_Affected tissues (F_OTU526, F_OTU503, F_OTU575 –*Phellinus gilvus,* F_OTU11 –*Candida norvegica*). Finally, there were OTUs more related to tissues from diseased sea stars (both, NON_Affected and Affected), than to samples (Healthy) coming from healthy individuals; e.g., F_OTU509 – *Aspergillus austroafricanus* (Fig. S3B and Table S2).
